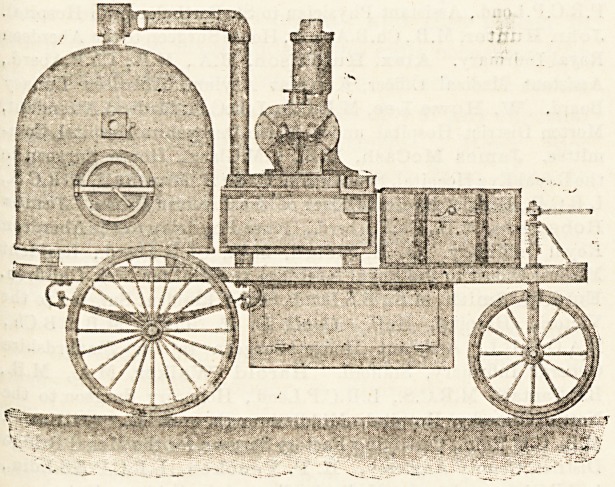# Practical Departments

**Published:** 1904-05-21

**Authors:** 


### PRACTICAL DEPARTMENTS.

### CLAYTON PORTABLE FUMIGATOR.

The hindrances which exist to the satisfactory disinfection of buildings and rooms are well known; the chief of these is the difficulty in obtaining a gaseous disinfectant sufficiently powerful and penetrating to satisfy bacteriological tests without having an injurious action on perishable articles. Accumulated experience has shown the advantages for this purpose of sulphurous oxide gas obtained by burning sulphur. At the same time there are essential defects in this method as it is usually employed, by burning sulphur in an open dish, all air outlets from the room being as nearly as possible closed. In the first place, sulphur will not burn in an atmosphere containing more than about 5 per cent. of the products of its own combustion, and for rapid and efficient disinfection higher percentages than this are desirable. A further objection is that the heated gases are apt to condense on the cooler surfaces of the furniture of the room, and to bleach or otherwise damage them. These obstacles have been overcome by the Clayton Fire Extinguishing and Ventilating Company, Limited, 22 Craven Street, Strand, who have introduced an apparatus by means of which high percentages of sulphurous anhydride at the normal temperature can be rapidly introduced into a chamber to be disinfected. The germicidal action of the sulphur gas can thus be more than doubled, while the danger of damaging oil-paintings and so forth is said to be actually lessened, as no condensation occurs. Hitherto the Clayton Fire Extinguishing and Ventilating Company have been mainly concerned with the disinfection of ships, for which they provide a single instalment for purposes of disinfecting, fire extinguishing, and ventilating. They have lately designed, however, a portable fumigator for the use of hospitals and other institutions, as well as for private house disinfection.

The apparatus, which is shown in the illustration, consists of (1) a large, dome-shaped generating chamber, in which the sulphur is burnt; (2) a blower, which draws air through the generator, and through (3) a cooler, by which it is reduced to a normal temperature, and then forces it through the (4) discharge pipe leading into the room which is to be disinfected. There is also a (5) return pipe which connects the room with the generator. As sulphur dioxide is heavier than air the return pipe should communicate with the upper part of the infected chamber while the discharge pipe opens near the floor. At the commencement of the disinfecting process the air supplied to the generator is conveyed by the return pipe from the room which is being treated. But as soon as the atmosphere in the room contains about 2½ per cent. of sulphur gas the return pipe is shut off by closing a trap, and, by opening a second trap, the generator is thenceforward supplied with air from the outside. The supply of sulphur gas is continued until the atmosphere of the room contains about 10 per cent., when the blower is stopped (a 3 per cent. vapour is said to be sufficient for the destruction of vermin), and the gas allowed to remain in the room from 4 to 12 hours. Penetration tests, both by litmus and by bacteriological cultures, have proved highly satisfactory, as might be expected from the high percentage of sulphur dioxide that is used. This percentage can be easily and rapidly determined at any time by means of a special burette. This consists of a graduated glass cylinder which is filled with atmosphere from the room which is being treated by means of a small piece of tubing passed through the key-hole or other small aperture. Water is then slowly passed into the tube until no more gas can be absorbed. As the sulphur dioxide is readily absorbed by water, the diminution in volume of the gases in the tube after the addition of water represents the amount of sulphur dioxide present. This can be seen at a glance by reading off the level of the added water from the graduated scale, which gives the percentage.

Altogether, as the result of our inquiry, we are of opinion that the Clayton Portable Fumigator offers the most reliable method of general disinfection, and should prove of great value to the public departments, as well as to hospitals, asylums, and other institutions, where the need for dealing with infected apartments is likely to arise.

## Figures and Tables

**Figure f1:**